# Effect of Thermal Treatment on Crystallinity of Poly(ethylene oxide) Electrospun Fibers

**DOI:** 10.3390/polym11091384

**Published:** 2019-08-23

**Authors:** Martina Polaskova, Petra Peer, Roman Cermak, Petr Ponizil

**Affiliations:** 1 Department of Polymer Engineering, Faculty of Technology, Tomas Bata University in Zlín, Vavreckova 275, 760 01 Zlín, Czech Republic; 2 Centre of Polymer Systems, Tomas Bata University in Zlín, Trida Tomase Bati 5678, 760 01 Zlín, Czech Republic; 3 Institute of Hydrodynamics of the Czech Academy of Sciences, Pod Patankou 5, 166 12 Prague 6, Czech Republic; 4 Department of Physics and Materials Engineering, Faculty of Technology, Tomas Bata University in Zlín, Vavreckova 275, 760 01 Zlín, Czech Republic

**Keywords:** PEO nanofibers, crystallinity, electrospinning, wide-angle X-ray diffraction, thermal treatment, thermooxidation

## Abstract

Post-process thermal treatment of electrospun fibers obtained from poly(ethylene oxide) (PEO) water and methanol solutions was examined. PEO fibers from methanol solution showed larger diameters as observed by scanning electron microscopy. Fibers both from water and methanol solutions exhibited a significant dimensional stability and surface cracking during the specific exposure time after thermal treatments at 40, 50, and 60 °C. Changes in crystallinity after the thermal treatment were studied by wide-angle X-ray diffraction. The kinetics of secondary crystallization were positively influenced by the as-processed level of the amorphous phase and temperature of thermal treatment. Samples treated at 60 °C were degraded by thermooxidation within the time.

## 1. Introduction

Poly(ethylene oxide) (PEO) is a nontoxic highly crystalline polymer with a glass transition temperature of approximately −50 °C, promising extensive chain flexibility [1]. It can be dissolved in various organic solvents, such as methanol or ethanol, and water, which classifies it as an easily spinnable material. Electrospinning is one of the processing methods enabling the production of micro-, submicro-, and nanofibers from polymer solutions and melts using electric forces [2,3,4]. Electrospinning of PEO solutions at room temperature has been thoroughly investigated, providing many research studies describing PEO nonwoven webs with various fiber sizes and molecular orientations depending on the chosen processing parameters [5,6], solvents [7], and solution properties [8].

PEO often serves as a carrier liquid facilitating the electrospinning process of some polymers, such as chitosan or cellulose [9]. These nanofibrous membranes perform many beneficial properties, including a high pore density and surface area. Therefore, they may be employed in diverse applications, such as tissue scaffold, wound dressing, or antibacterial membranes [9,10]. However, nanofibrous membranes lack mechanical strength, which could be enhanced by introducing interfiber bonding after specific treatments. Generally, a thermal treatment is used to bond the electrospun nanofibers. Thermal treatment of nanofibers to improve the crystallinity and mechanical properties of various polymers, such as poly(vinylidene fluoride), poly(4-methyl-1-pentene), polyester, and PEO, has recently been reported [11,12,13,14]. For instance, Bao et al [14] studied the morphology of PEO nanofibrous membranes either with or without multiwall carbon nanotubes thermally bonded at temperatures ranging from 60 to 64 °C. To the authors’ knowledge, the thermal-induced morphology of pure PEO nanofibers has not been examined yet.

The crystallinity and molecular orientation of electrospun fibers are normally significantly lower if compared with samples prepared by common processing technologies (molding or film casting) [15]. Macroscopic alignment of fibers was gained either by a rotating mandrel collector [1] or by counter-electrode plates’ integration [16,17]. Nonetheless, macroscopic alignment may not always be connected with the orientation on the microscopic level and the degree of crystallinity remains almost the same as without the alignment. The structure’s formation during the electrospinning process is indeed a complex issue. The most critical parameters affecting the crystallization process include the degree of chain orientation/relaxation and the rate of solvent evaporation [18].

Post-processed thermal treatment seems to be the proper technique, leading both to evaporation of the residual solvent and an increase of the crystalline phase [19]. A reduction in the interlamellar distance, also known as lamellar thickening, is typical for this phenomenon. Nevertheless, amorphous and crystalline phase rearrangement can be accompanied with the thermooxidation process in the solid state [20]. If compared to pure hydrocarbon polymers, such as poly(ethylene) and poly(propylene), PEO is more sensitive to thermal oxidation [21]. In this context, scission of the chains during this process also induces morphological changes that can be reflected in the overall degree of crystallinity. Thus, the proper temperature–time condition for post-process thermal treatment should be established and was examined in this paper.

## 2. Materials and Methods

### 2.1. Materials

Polyethylene oxide (PEO) of *M*_w_ = 300,000 g/mol was purchased from Sigma Aldrich (St. Louis, MO, USA). It was dissolved in distilled water and methanol (9 wt.%). PEO solutions were stirred with a mixing rate of 250 rpm for 48 h using a magnetic stirrer (Heidolph MR Hei-Tec, Schwabach, Germany) with a teflon-coated magnetic cross at 25 °C for water solutions and 35 °C for methanol solutions.

### 2.2. Characterization of the Solution

A rotational rheometer Physica MCR 501 (Anton Paar, Graz, Austria) with the concentric cylinder arrangement with inner and outer diameters of 26.6 and 28.9 mm, respectively, was used to determine the rheological properties of PEO solutions at 25 °C. Rheological measurements of the polymer solutions were performed in steady (a range of shear rates from 0.01 to 300 s^−1^) and oscillatory (at a constant strain of 1% and with a frequency ranging from 0.1 to 100 s^−1^) modes.

### 2.3. Electrospinning

Nanofibrous webs were created by the laboratory device [22], which consisted of a high-voltage power supply (Spellman SL70PN150, Hauppauge, NY, USA), carbon-steel stick of a diameter of 10 mm, and motionless flat-metal collector. The electrospinning process was performed from a drop of polymer solution (0.2 mL) placed on the tip of a steel stick at a voltage of 25 kV with the tip-to-collector distance fixed at 200 mm at 20 ± 1 °C with a relative humidity of 38 ± 3%.

### 2.4. Thermal Treatment of Nanofibrous Web

In order to avoid any structural transformations of the prepared samples, electrospun webs were cooled to –75 °C (a significantly lower temperature than PEO *T*_g_ of –54 °C) immediately after the electrospinning process and stored at this temperature for further experiments. Afterwards, PEO nanofibrous webs were exposed to thermal treatments at various temperatures of 40, 50, and 60 °C for a specified period of time.

### 2.5. Characterization of Nanofibrous Web

Characteristics of nanofibrous webs were evaluated using a Vega 3 high resolution scanning electron microscope (Tescan, Brno, Czech Republic). A conductive coating layer was applied in advance. To determine the mean fiber diameter, Adobe Creative Suite software was used. In total, 300 measurements were executed in three different images.

Crystallinity of the prepared samples was characterized by wide-angle X-ray diffraction (WAXD). Diffractograms were recorded by a Philips diffractometer (XPertPRO, Almelo, The Netherlands) equipped with a hot stage. Measurements were completed in a reflection mode in the 2θ range of 10 to 35° with a nominal resolution of 0.03° at selected temperatures and a quasi-logarithmic sampling frequency. Every test was performed in duplicate to enhance the repeatability.

## 3. Results and Discussion

The diameter of nanofibers depends on various factors: (i) Polymer characteristics (molecular weight and topology of polymer macromolecules), (ii) solvent characteristics (vapor pressure, surface tension, viscosity), (iii) solution properties (concentration, viscosity and elasticity of solution), and (iv) electrospinning parameters (electric field strength, tip-to-collector distance, temperature, and humidity). The basic characteristics of the solvents are summarized in Table 1. Identical PEO was used for water and methanol solutions. Table 2 shows the rheological behavior of the solutions and resulting fiber diameters. As can be seen, the viscosity of methanol/PEO solutions was lower compared with water/PEO solutions, although the concentration was maintained on the same level. However, electrospun fibers obtained from methanol solutions exhibited significantly larger diameters. Thus, in this case, the main parameter controlling the fiber thickness is the evaporation kinetics of a specific solvent represented by the vapor pressure; ejected jets from the Taylor cone can be drawn in the electric field until evaporation of the solvent and fiber solidification. In addition, solvent quality can be derived using Hansen solubility parameters, indicating the degree of interaction between a polymer and a solvent [23]. The solubility parameter of PEO is 10.5 (cal cm^−3^)^1/2^, methanol is 29.6 (cal cm^−3^)^1/2^, and water is 47.8 (cal cm^−3^)^1/2^ [23,24]. Due to smaller differences in the total solubility parameters, methanol is more suitable as a solvent for PEO than water.

**Figure 1:**
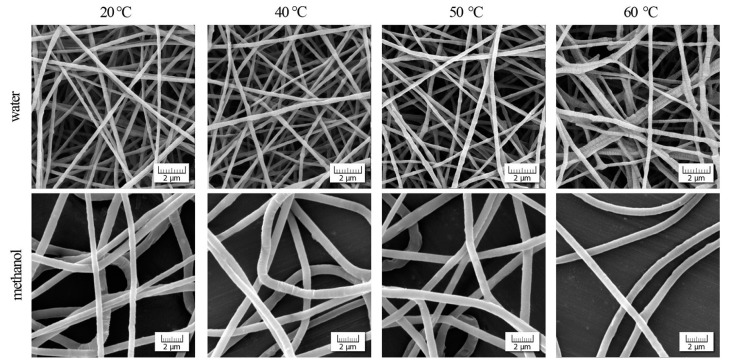
Effect of temperature on morphology of fibers obtained from PEO solutions.

Figure 1 shows the micrographs of electrospun fibers after four-hour treatments at selected temperatures. As can be seen, the initial smooth surface and diameter of the fibers are almost intact after the thermal treatment in a provided interval. However, the surface of the fibers obtained from the water solution tends to roughen, break, and re-join at a temperature of 60 °C as visible in the upper-right corner of the micrograph. This indicates thermodynamic instability of the fibers. Surface roughening can be ascribed to the chemi-crystallization process [25]. This phenomenon has been described and explained for several polymers and relates to a chain scission on the surface layer. Released macromolecular segments can be incorporated into the already existing crystallites, resulting in surface shrinkage and cracking, particularly on the lamellae borders.

**Figure 2:**
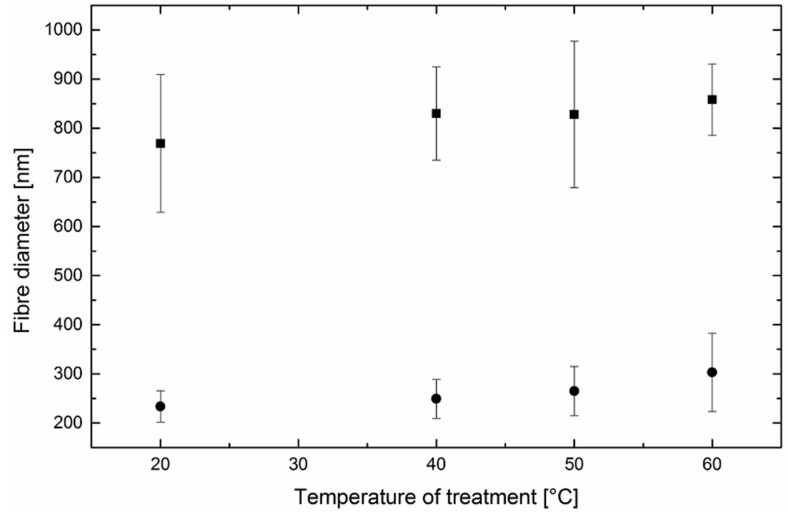
Effect of temperature on the diameter of fibers obtained from PEO solutions. Square symbols denote methanol solutions and circles denote water solutions.

The structure of thermal-treated nanofibrous webs consists of well-identified separated fibers, which enables a clear image analysis and calculation of the fiber diameters. Figure 2 depicts the mean fiber diameter and corresponding standard deviation as a function of the temperature of the thermal treatment. As no extensive shrinkage of the oriented amorphous portion occurred, it can be concluded that the diameter variation is statistically insignificant. Although electrospun fibers generally exhibit a high amount of the amorphous phase [26], the level of crystallinity was sufficiently high enough to dimensionally stabilize the nanofibrous webs in this experiment.

**Figure 3:**
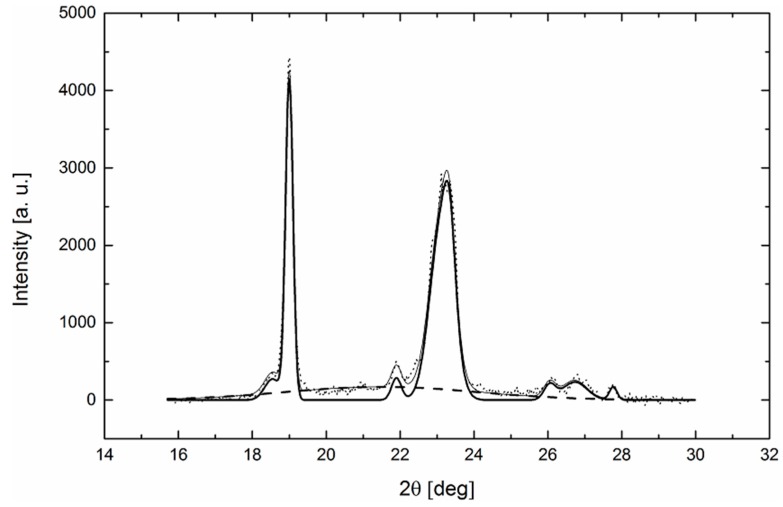
Diffractogram of nanofibrous PEO sample separated into individual crystalline peaks (solid thick line) and an amorphous halo (dashed line). The dotted line shows the experimental data and the solid thin line represents the amorphous and crystalline part fitted together.

Crystallinity development in nanofibers after thermal treatment can be examined non-destructively by wide-angle X-ray diffraction. Typical X-ray patterns of PEO are illustrated in Figure 3. Sharp reflections in diffractograms correspond to PEO crystallites. Furthermore, a wide diffused halo is evidence of the amorphous phase. To determine crystallinity precisely, diffractograms have to be separated into individual crystalline peaks and an amorphous halo. In this case, each crystalline peak and amorphous halo was fitted by an individual Gauss function. Finally, crystallinity of the samples was calculated as a ratio between the sum of crystalline peak areas and the area of the whole X-ray pattern.

**Figure 4:**
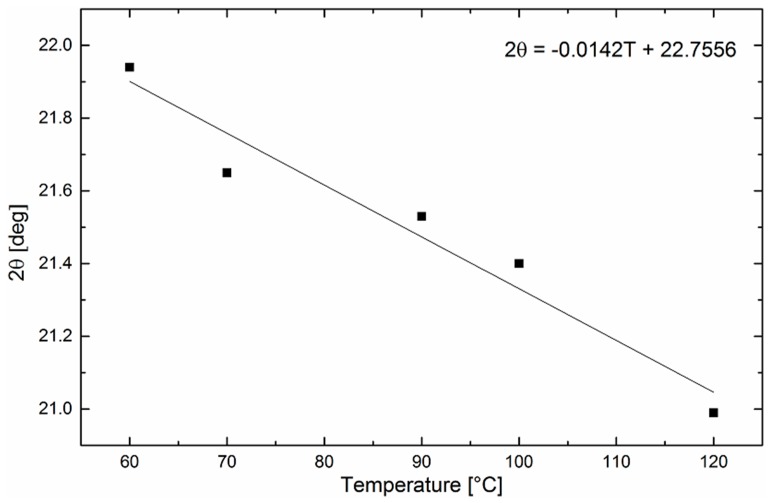
Position of an amorphous halo as a function of the temperature of melted PEO.

While crystalline peaks can be unambiguously identified, the maximum of the amorphous halo varies with the temperature and must be evaluated experimentally. For these purposes, X-ray patterns were measured in the melt state at various temperatures and the maximum of the individual amorphous halo at a specific temperature was assessed [27,28]. In Figure 4, the position of the maximum amorphous halo as a function of temperature is shown. It can be stated that under specific experimental conditions, the maximum of the amorphous halo is a linear function of the temperature. Then, the maximum position can be extrapolated to the solid state, i.e., at temperatures below 60 °C, and the diffractogram can be separated into individual crystalline peaks and an amorphous halo.

**Figure 5:**
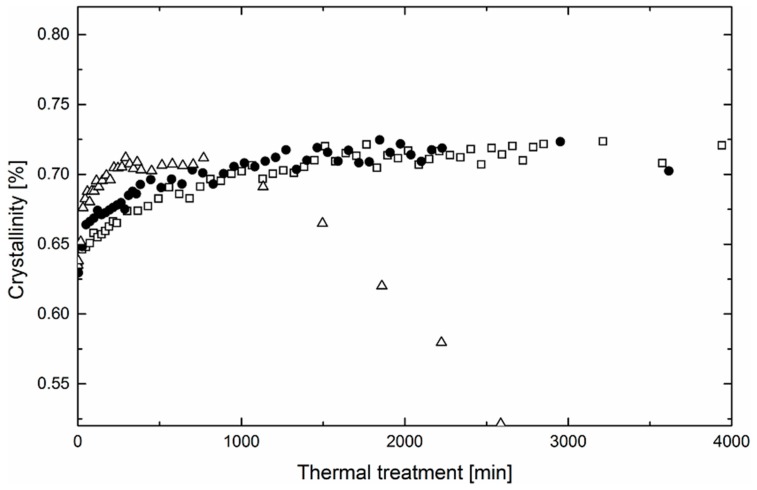
Evolution of crystalline structure of nanofibrous webs prepared from water/PEO solution at three different temperatures in time (squares denote 40 °C, circles 50 °C, and triangles 60 °C).

**Figure 6:**
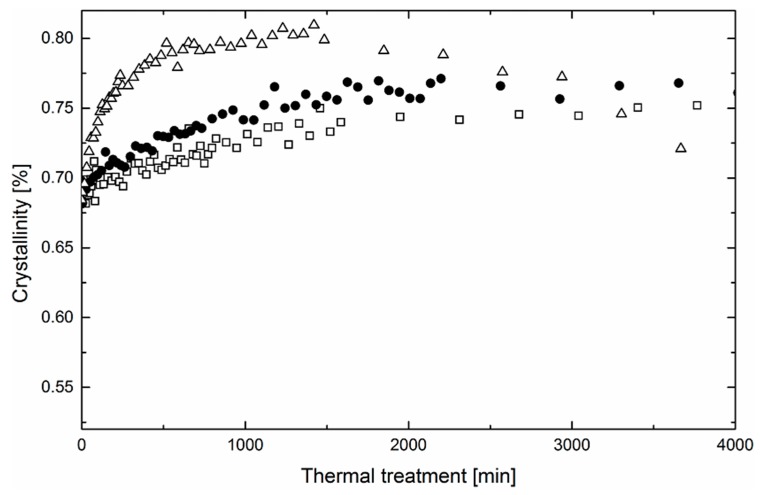
Evolution of crystalline structure of nanofibrous webs prepared from methanol/PEO solution at three different temperatures in time (squares denote 40 °C, circles 50 °C, and triangles 60 °C).

Figure 5 and Figure 6 show the evolution of crystallinity of the nanofibrous webs after the thermal treatment at various temperatures. As can be seen, the initial crystallinity of nanofibers is defined by the type of electrospun solution. Fibers prepared from methanol solution have a crystallinity of 5%, higher than water solution fibers. This may be on account of the surface irregularity and solvent quality. It is expected that the fibers’ surface solidifies extremely fast, which leads to a higher level of structural defects if compared with the core of the fiber [29]. Consequently, the overall crystallinity of the fibers depends on the surface/volume ratio. Thinner fibers from the water solution are more amorphous. Furthermore, the solvent quality also plays an important role in the overall crystallinity of the fibers obtained from solution. It has been reported that the solvent considerably affects the mobility of dissolved macromolecular segments, manifesting itself in improved crystallization kinetics and crystallite development [30].

What is common for fibers prepared from water and methanol solutions is the monotonic growth of crystallinity in the initial phase of the process of thermal annealing. The kinetics of the process depends on the temperature and as-processed crystallinity. At the beginning, the crystallinity of fibers prepared from water solutions grows significantly faster than that of fibers prepared from methanol and the process is positively influenced by the annealing temperature. Consequently, the rate of secondary crystallization in PEO is controlled by the mobility of the polymeric chains, which is generally higher in the amorphous phase.

After a certain time, crystallinity changes induced by the thermal treatment should reach the maximum. However, in the experiment, the maximum was reached in the samples prepared from both methanol and water solutions only at the temperature of 60 °C. Regarding the temperatures of 40 and 50 °C, crystallinity maxima can be expected after longer periods of thermal treatment. It is interesting to note that the crystalline portion of the samples thermally treated at 60 °C is not stable and with a prolonged process, its level decreases even below the as-processed values. This indicates a competing process of degradation proceeding mutually during the thermal treatment. It has been reported that PEO is sensitive to thermooxidation even at relatively low temperatures. Morlat et al. states that thermooxidative changes can be observed after thermal treatment of PEO at 50 °C [21]. The overall kinetics of the degradation are controlled by oxygen diffusion. From the morphology point of view, oxygen diffusion dominates in amorphous regions. As a consequence, massive thermooxidative changes manifested by a decrease of crystallinity are faster in the fibers prepared from water solution.

## 4. Conclusions

The experimental results of this study have shown the following conclusions for PEO electrospun fibers:

1. Electrospun PEO fibers processed from methanol solution possess higher diameters compared with fibers obtained from water. It respects the evaporation kinetics of a solvent.

2. PEO fibers prepared from methanol and water solutions by electrospinning are dimensionally stable even during thermal treatment close to their melting temperature. 

3. As a consequence of chemi-crystallization, the fiber surface is roughened after a specific period of thermal treatment.

4. Solvent quality positively influences the as-processed crystallinity of PEO electrospun fibers, which is manifested by the higher crystalline portion in fibers prepared from methanol solution.

5. The crystalline phase increment during secondary crystallization is positively impacted by the as-processed crystalline level of PEO electrospun fibers. Similarly, the temperature of the thermal treatment accelerates secondary crystallization in the observed temperature interval.

6. At a prolonged time of thermal treatment at 60 °C, massive thermooxidative degradation of both types of electrospun PEO fibers is manifested by a decrease in crystallinity.

## Figures and Tables

**Table 1:** Characteristics of the applied solvents.

Solvent	Viscosity [mPa.s] at 25 °C	Relative Permittivity	Specific Conductivity [S/m]	Surface Tension [mN/m]	Vapor Pressure [mm.Hg]
Water	0.89	80.1	5.5 × 10^−6^	72	175
Methanol	0.59	32.7	1.5 × 10^−7^	22.1	100

**Table 2:** Rheological properties of PEO solutions (9 wt.%) measured at 20 °C and the mean diameter of fibers.

Solvent	Shear Viscosity [Pa.s]	Phase Angle [°]	Mean Diameter [nm]	Standard Deviation [nm]
Water	5.4	57.6	200	30
Methanol	1.3	61.1	768	140
